# Rhamnolipid exhibits anti-biofilm activity against the dermatophytic fungi *Trichophyton rubrum* and *Trichophyton mentagrophytes*

**DOI:** 10.1016/j.btre.2020.e00516

**Published:** 2020-08-19

**Authors:** Suparna Sen, Siddhartha Narayan Borah, Arijit Bora, Suresh Deka

**Affiliations:** a Environmental Biotechnology Laboratory, Resource Management and Environment Section, Life Sciences Division, Institute of Advanced Study in Science and Technology, Vigyan Path, Paschim Boragaon, Garchuk, Guwahati, 781035, Assam, India; b Centre for the Environment, Indian Institute of Technology Guwahati, North Guwahati, Guwahati, 781039, Assam, India; c Department of Bioengineering and Technology, Institute of Science and Technology, Gauhati University, Gopinath Bordoloi Nagar, Guwahati, 781014, Assam, India

**Keywords:** *Trichophyton* spp., Rhamnolipids, Anti-biofilm activity, Ultramicroscopy, Biosurfactant

## Abstract

Dermatophytes are responsible for a majority of fungal infections in humans and other vertebrates, causing dermatophytosis. Treatment failures are often associated with biofilm formation, making dermatophytes resistant to antifungals. In this study, effects of a rhamnolipid (RL-SS14) produced by *Pseudomonas aeruginosa* SS14 on planktonic cells of *Trichophyton rubrum* and *Trichophyton mentagrophytes*, their biofilm formation, and disruption of mature biofilms were assessed. The composition of RL-SS14 was analysed using FTIR, HPLC-ESI-MS, and GC–MS. Minimum inhibitory concentrations against the planktonic forms of *T. rubrum* and *T. mentagrophytes* were 0.5 mg/mL and 0.125 mg/mL, respectively. Crystal-violet (biofilm biomass) and safranin (extracellular matrix) staining revealed that RL-SS14 significantly inhibited biofilm formation and also reduced preformed biofilms in a dose-dependent manner. Microscopic visualization of treated biofilms via SEM, AFM, and CLSM revealed marked morphological damage, cell death, and reduced extracellular matrix. The results indicate the potential of RL-SS14 as an anti-biofilm agent against dermatophytes.

## Highlights

• First report of the activity of rhamnolipid (RL) against dermatophytic biofilms.
• RL acts as an anti-biofilm agent against *T. rubrum* and *T. mentagrophytes*.
• RL inhibits biofilm formation as well as disrupts mature biofilms.
• Marked reduction of biomass and extracellular matrix of biofilms on RL exposure.
• RL was characterized using FT-IR, HPLC-ESI-MS, and GC–MS.

## 1 Introduction

Dermatophytes, a group of filamentous fungi, can invade and infect keratinized tissues of humans and animals, causing dermatophytosis. It is one of the most common superficial fungal infections affecting about a quarter of the global population [1]. Even though it does not lead to mortality, it causes significant morbidity apart from posing a critical public health problem, especially in tropical and subtropical developing countries like India. In these regions, the hot and humid climate favors the acquisition and maintenance of the disease [2]. In several parts of the world, about 90 % of cases of chronic dermatophytosis have been attributed to *Trichophyton* spp. [3]. Again, treatment failure with antifungals is increasingly reported, especially in patients infected with *T. rubrum*, which, among other factors, can largely be attributed to biofilm formation [4]. Biofilms are of critical importance because they are implicated in a significant proportion of all clinical infections interfering with medicinal therapy [5]. Biofilms are incredibly resistant to most of the clinically used antimicrobials with inhibitory concentrations being 100-fold higher than that needed to inhibit planktonic cells [6]. Contributing factors for such resistance include structural complexity, extracellular matrix (ECM), intrinsic metabolic heterogeneity, and biofilm-associated up-regulation of efflux pump genes [7]. The biofilm-forming ability of dermatophytes was first suggested by Burkhart et al. [8] in dermatophytoma associated with onychomycosis. The architecture and growth characteristics of dermatophytic biofilms (*Trichophyton* spp., *Microsporum* spp.) have only been recently determined [[9], [10], [11]]. Further, filamentous fungal biofilms are structurally complex and less-studied, making treatment difficult [11]. Therefore, there is an unfulfilled need for newer antifungals or approaches for treating recalcitrant dermatophytosis to provide an effective, novel, and non-toxic alternative to conventional therapeutics.

Biosurfactants (BS) or microbially derived surfactants are amphiphilic molecules of bacterial, fungal, or yeast origin, that exhibit excellent surface-activity, structural diversity, and environmental compatibility [12]. BS promotes the uptake of poorly soluble substrates, heavy metal binding, modulates the immune response, and act as antimicrobial compounds [13]. Application of BS as anti-biofilm agents has been explored widely in recent times against several bacterial and fungal species [[14], [15], [16]]. Under specific testing conditions, BS has been reported to be efficacious than many traditional inhibitory and disruptive strategies against biofilms [17,18]. In general terms, BS are thought to achieve the anti-biofilm effect through alteration of surface energy and surface wettability [19]. Glycolipids are BS consisting of a carbohydrate linked to an aliphatic or hydroxy-aliphatic acid. Among them, the best known are rhamnolipids (RLs), which includes di- or mono-rhamnose sugars linked to a hydroxy fatty acid chain. RLs are among the most studied groups of BS in various fields; nevertheless, they are under-represented as anti-biofilm agents [20]. In nature, RLs are always produced as combinations of different homologs [21], and evidence shows that individual molecules can exert distinct biological effects [22]. RLs are effective against biofilms of *Bordetella bronchiseptica, Bacillus pumilus*, *Streptococcus salivarius*, *Staphylococcus* spp., *Candida tropicalis*, and *Yarrowia lipolytica* [16,19,23]. Despite RLs being potent antimicrobial agents, reports documenting their use against fungal biofilms are sketchy, with the majority of the studies being limited to *Candida* biofilms [24]. We have earlier investigated the antifungal activities of RL against the planktonic forms of *T. rubrum* and obtained promising results [25]. Hence, to further the information on the antifungal properties of RL, this study reports the effects on the biofilms formed by *T. rubrum* and *T. mentagrophytes*. To the best of our knowledge, the antibiofilm efficacy of RL against dermatophytes is unreported.

## 2 Materials and methods

### 2.1 Microorganisms, growth conditions, and production of rhamnolipid

*Trichophyton rubrum* MTCC 8477 and *Trichophyton mentagrophytes* NCCPF 800049 procured from Microbial Type Collection and Gene Bank (MTCC), IMTECH, India, and National Culture Collection of Pathogenic Fungi (NCCPF), ICMR, India respectively were used as pathogens for the anti-biofilm studies. The fungal strains were grown in Sabouraud dextrose broth/agar plates (SDB/SDA, HiMedia, India) at 25 ± 2 °C for routine use and maintained in 30 % (v/v) glycerol stocks at -80 °C. The biofilm-forming abilities of both the strains were confirmed before performing the study (Fig. S1 of Supplementary data).

*Pseudomonas aeruginosa* SS14 (GenBank Accession no: KC866140, [26]) was used for the production of rhamnolipid (RL-SS14). The bacterial inoculum was prepared in nutrient broth (NB, HiMedia) and incubated at 35 °C for 24 h under the agitation of 150 rpm. From this overnight culture, 5 ml inoculum was transferred to mineral salt medium (MSM) containing 10 % (w/v) carbon substrates (glucose, glycerol, mannitol, or molasses) for RL production and incubated under similar conditions for 48 h. The surface tensions of the culture media were measured using a tensiometer (K11, Kruss, Germany). The RLs were recovered from the cell-free culture broth after 48 h by solvent extraction using ethyl acetate. Crude RL-SS14 was then column purified with silica gel column chromatography containing activated silica gel 60–120 chloroform slurry. A detailed description of the MSM composition, cultivation conditions for RL production by *P. aeruginosa* SS14, extraction, and purification of RL-SS14 have been described in our previous study [26].

### 2.2 Antifungal susceptibility testing

Antifungal activities of column purified RL-SS14 produced with different substrates (glucose, glycerol, mannitol, or molasses) were evaluated to determine the most efficient carbon substrate based on the highest inhibition against the tested pathogens. The minimal inhibitory concentration (MIC) against the planktonic cells was evaluated via broth microdilution assay as per the Clinical and Laboratory Standards Institute (CLSI, M38-A2) guidelines for filamentous fungi [27]. For inoculum preparation, the fungal strains were grown in SDA plates until conidia formation. Stock inoculum suspensions of each strain were obtained by covering the fungal colonies with saline and gently transferring the conidial suspension to sterile centrifuge tubes. Conidial suspensions were adjusted at 2 × 10^5^ colony forming units (CFU)/mL microscopically using a haemocytometer with RPMI-1640 (Sigma-Aldrich, USA) medium supplemented with l-glutamine and buffered to pH 7.0 with 3-[N-morpholino] propane sulfonic (MOPS) acid (Sigma-Aldrich, USA) [2]. Double strength stock solutions of RL-SS14, standard RL (R-95; Sigma-Aldrich, USA), and reference antifungal terbinafine (TBF; Sigma-Aldrich, USA) were prepared in RPMI-1640 medium. One hundred microliter of the conidial suspensions were added to the wells of the microtiter plates (Tarsons, India) containing 100 μL of serially double diluted stock solutions of RL-SS14, R-95, or TBF to yield the final concentrations (2.0 to 0.003 mg/mL). Then the plates were incubated at 28 °C for 96 h. Wells without RL-SS14, R-95, or TBF containing the spore suspension served as growth control. Sterility controls with the media, but without the fungi were also included. The absorbance was measured in a Multimode Reader (Thermo Scientific, USA) at 600 nm and the inhibition (%) was calculated as:

$$\;Inhibition\;\left(\%\right)=\frac{\left({OD}_{control}-{OD}_{treated}\right)}{{OD}_{control}}\times 100$$

MIC values corresponded to the lowest concentration that completely inhibited the growth of *T. rubrum* and *T. mentagrophytes* after 96 h.

The minimum fungicidal concentration (MFC) was determined by transferring aliquots from wells showing no visible growth onto SDA plates, followed by incubation at 25−28 °C for seven days. MFC was defined as the lowest concentration that resulted in no visible fungal growth/colonies [28].

### 2.3 Selection of suitable carbon source and characterization of the most efficient rhamnolipid

Based on the initial antifungal susceptibility testing, RL-SS14 exhibiting the highest inhibitory activity against the pathogens under study was selected as the most efficient RL and used for further studies. The corresponding carbon substrate was chosen as the most suitable carbon source. The selected RL-SS14 was subsequently characterized by FT-IR, GC–MS, and HPLC-ESI-MS analyses. FT-IR was performed in attenuated total reflectance (ATR) mode using a Nicolet 6700 FTIR System (Thermo Scientific, Waltham, MA) at a resolution and wavenumber accuracy of 4 and 0.01 cm^−1^, respectively, and 64 scans with correlation for atmospheric CO_2_.

Fatty Acid Methyl Esters (FAMEs) of the sample was analyzed in a GC/MS TQ8030 system (Shimadzu, Japan) as per the protocol previously described without any modifications to identify the fatty acid side chains present in RL-SS14 [29]. HPLC-ESI-MS was performed using a 1260 Infinity LC attached to a 6410 Triple Quad MS (Agilent Technologies, USA) having an electrospray ionization (ESI) interface for the chromatographic separation and identification of the structural congeners in RL-SS14 [26].

### 2.4 Inhibition of biofilm formation

The strains were initially grown on SDA plates until sporulation. The spore inoculum suspension was prepared at a concentration of 2 × 10^6^ CFU/mL for *T. rubrum* and *T. mentagrophytes* in RPMI-1640 medium. The inhibitory effect of RL-SS14, R-95, and TBF on biofilm formation was quantified according to a procedure previously described (co-incubation assay) [2]. Briefly, 100 μL of serially two-fold diluted solutions of RL-SS14, R-95, or TBF prepared in RPMI-1640 were added to sterile 96-well polystyrene microtiter plates. Then, the wells were seeded with 100 μL of fungal inocula (final concentration 1 × 10^6^ CFU/mL) and the plates were incubated at 28 °C for 96 h. After incubation, the wells were washed with phosphate-buffered saline (PBS) to remove planktonic cells. Fungal suspension in the medium without RL-SS14, R-95, or TBF served as the negative control and the broth without the fungal strains was included as sterility control. TBF treated fungal suspensions served as positive control. The sessile cells and the extracellular matrix (ECM) were quantified with crystal-violet (CV) assay and safranin staining, respectively, as described previously [9]. The absorbance of the bound dye was measured in a multimode reader and represented as biofilm formation (%).

$$Biofilm\;formation\;\left(\%\right)=\;\frac{{OD}_{treated}\;}{{OD}_{control}\;}\;\times 100$$

#### 2.4.1 Crystal-violet staining

After emptying the wells, they were thoroughly washed with PBS to remove unattached cells. Subsequently, the biofilms formed in the wells were stained with 100 μL of 0.5 % CV solution for 15 min. Excess stain was removed by washing with sterile water. Then, 100 μL ethanol (95 % v/v) solution was added to each well to decolorize the biofilms. The complete removal of CV was ensured by homogenizing the ethanol solution with a pipette (∼ 1 min). The contents were then transferred to a fresh plate, and the absorbance was measured at 570 nm.

#### 2.4.2 Safranin staining

Safranin stains polar structures, e.g., fungi cell wall components and polysaccharides in the ECM [30]. Safranin staining was performed to quantify the extracellular matrix produced by the biofilm, as described previously [9]. The biofilms were washed with PBS and stained with 100 μL safranin solution for 15 min. The wells were aspirated and cleaned thoroughly, then subjected to the measurement of the absorbance at 492 nm.

### 2.5 Biofilm dispersal

*T. rubrum* and *T. mentagrophytes* biofilms were formed in 96-well flat-bottom microtiter plates as described previously [9]. One hundred microliters of the microconidial suspensions (1 × 10^6^ CFU/mL in RPMI 1640) were inoculated in the wells except for the sterility control and incubated at 28 °C for 96 h without shaking for biofilm formation. Each well was then decanted to remove the planktonic cells. The sessile cells were washed gently with PBS. RPMI-1640 media with RL-SS14, R-95, or TBF (positive control) at different concentrations (0.25 × MIC, 0.5 × MIC, 1 × MIC, 2 × MIC) were added to the wells and incubated overnight at 28 °C to determine the biofilm dispersal ability of the test compounds measured as biofilm (%) with CV or safranin staining as described in the previous section.

$$Biofilm\;\left(\%\right)=\;\frac{{OD}_{treated}\;}{{OD}_{control}\;}\;\times 100$$

### 2.6 Imaging

*T. rubrum* and *T. mentagrophytes* biofilms were formed on sterile coverslips placed in 24 well-plates filled with RPMI-1640 medium for 96−120 h [9]. The coverslips were thoroughly washed with PBS and then treated with 2 × MIC concentrations of RL-SS14 (1.0 mg/mL for *T. rubrum* and 0.25 mg/mL for *T. mentagrophytes*) and TBF (0.03 mg/mL for *T. rubrum* and 0.062 mg/mL for *T. mentagrophytes*) for 24 h. Untreated samples were kept as negative controls. For ultramicroscopic studies, the coverslips were recovered post-treatment and washed with PBS followed by overnight fixation in 2.5 % (v/v) glutaraldehyde in PBS (0.1 M, pH 7.5) at 4 °C. After washes with PBS to remove any trace of the fixative, the samples were dehydrated sequentially in an increasing gradient of ethanol (30–100 %).

For Scanning Electron Microscopy (SEM), the dehydrated samples were mounted on stubs over carbon tapes and observed in a Field Emission Scanning Electron Microscope (FE-SEM, Σigma, Zeiss, Germany).

For Atomic Force Microscopy (AFM), the samples were visualized in an atomic force microscope (NTEGRA prima, NTMDT Technology, Russia) equipped with a silicon cantilever (spring constant – 0.03 N/m) in semi-contact mode (scan range – 100 × 100 × 5 μm^3^). For analysis of the images, Nova software version 1.1.0.1780 was used.

For Confocal laser-scanning microscopy (CLSM), treated and untreated fungal biofilms were stained with 100 μg/mL propidium iodide (PI; Sigma-Aldrich, USA) for 15 min, and viewed in a Leica TCS SP8 microscope with VIS Laser (Leica microsystems, Germany).

### 2.7 Statistical analysis

All the experiments were carried out in triplicates, and the anti-biofilm data were presented as mean ± standard deviation (SD). One-way analysis of variance (ANOVA) was used to compare the antibiofilm effect of the test samples, followed by pair-wise least significant difference (LSD) test for each treatment within each concentration, and p < 0.05 was considered significant. Statistical analyses were performed using the IBM SPSS Statistics 25.0, IBM Corp., Armonk, USA.

## 3 Results

### 3.1 Antifungal activity

The antifungal activities (MIC and MFC) of RL-SS14 produced using single substrates such as glucose, glycerol, mannitol, or molasses have been depicted in Table 1. MICs and MFCs` of R-95 against *T. rubrum* were found to be 0.25 and 0.5 mg/mL, respectively, whereas, for *T. mentagrophytes,* it was recorded at 0.062 mg/mL and 0.25 mg/mL, respectively. In the case of TBF, both MIC and MFC were found to be 0.015 mg/mL against *T. rubrum* and 0.031 mg/mL against *T. mentagrophytes*. For RL-SS14, it was observed that the antifungal activities varied based on the substrates used and the pathogens tested. MICs of the RL-SS14 ranged from 0.5 to 1.0 mg/mL and 0.125 to 0.5 mg/mL for *T. rubrum* and *T. mentagrophytes,* respectively. As compared to other carbon substrates, RL-SS14 produced utilizing glucose and glycerol exhibited superior activities against *T. rubrum* (MIC 0.5 mg/mL; MFC 1 mg/mL) and *T. mentagrophytes* (MIC 0.125 mg/mL; MFC 0.5 mg/mL), respectively. The MFCs were twice (*T. rubrum*) or four times (*T. mentagrophytes*) of the MICs, depicting the fungicidal potential of RL-SS14 against the pathogens.

**Table 1:** The antifungal activities of the rhamnolipids produced by *Pseudomonas aeruginosa* SS14 (RL-SS14) using different carbon substrates against *Trichophyton rubrum* and *Trichophyton mentagrophytes*. (MIC-Minimum inhibitory concentration, MFC-Minimum fungicidal concentration).

Carbon substrates	Antifungal activity (mg/mL)				
	*T. rubrum*		*T. mentagrophytes*		
	MIC	MFC		MIC	MFC
Glucose	0.5	1.0		0.25	1.0
Glycerol	1.0	>2.0		0.125	0.5
Mannitol	0.5	>2.0		0.25	1.0
Molasses	1.0	>2.0		0.5	2.0

### 3.2 Characterization of the most suitable rhamnolipid

Further experiments to determine the anti-biofilm activity against each pathogen were conducted with the RL-SS14 produced using the respective suitable carbon sources, i.e., glucose for *T. rubrum* and glycerol for *T. mentagrophytes*. The rhamnolipid produced using glucose has been previously characterized in detail and comprises of the mono-rhamnolipid congeners Rha-C_8_ and Rha-C_10_-C_10_ [31]. Hence, in this study, the detailed characterization has been performed only for the RL-SS14 produced using glycerol as the sole carbon source.

FT-IR analysis of RL-SS14 revealed the presence of functional groups similar to those of rhamnolipids. The −OH stretching of the hydroxyl group was detected at 3363 cm^−1^, while the C—H stretching of aliphatic groups of the lipidic side chains were detected at 2925 (asymmetric) and 2850 (symmetric) cm^−1^. The characteristic band of carbonyl groups (–C = O) for rhamnolipids was detected at 1732 cm^−1^. The peak at 1455 cm^−1^ corresponded to the in-plane bending of the O—H in carboxylic acid groups, while the peak at 1037 cm^−1^ represented the –C–O– stretching between the carbon atoms and hydroxyl groups within the rhamnose rings of rhamnolipid.

The FAME analysis by GC–MS revealed that RL-SS14 contained a total of 11 different 3-OH-Fatty Acids that were saturated, or involving monoenoic or dienoic unsaturations (Table 2, Figs. S2, S3 of Supplementary data).

**Table 2:** Composition of 3-hydroxy fatty acid methyl esters (FAMEs) in the rhamnolipid produced by *Pseudomonas aeruginosa* SS14 (RL-SS14) using glycerol as the carbon source. The analysis was carried out by GC–MS.

FAME	Retention time (min)	Abundance (%)	Molecular formula	Molecular weight	Component of Rhamnolipid
C_8:2_	5.27	0.62	C_9_H_14_O_3_	170.20	Rha-C_8:2_
C_8:0_	5.53	8.42	C_9_H_18_O_3_	174.23	Rha-C_8_-C_10_, Rha_2_-C_8_-C_10_, Rha_2_-C_8_-C_10:1_
C_9:2_	5.78	2.78	C_10_H_16_O_3_	184.23	Rha-C_9:2_
C_10:2_	5.99	0.11	C_11_H_18_O_3_	198.26	Rha-C_10:2_
C_10:1_	6.03	1.63	C_11_H_20_O_3_	200.27	Rha-C_10:1_, Rha_2_-C_8_-C_10:1_
C_10_	6.48	44.90	C_11_H_22_O_3_	202.29	Rha-C_8_-C_10_, Rha-C_10_-C_12:2_, Rha_2_-C_10_, Rha_2_-C_8_-C_10_, Rha_2_-C_10_-C_10_
C_12:2_	7.45	3.65	C_13_H_22_O_3_	226.31	Rha-C_12:2_, Rha-C_10_-C_12:2_, Rha_2_-C_12_-C_12:2_
C_12:0_	7.52	5.73	C_13_H_26_O_3_	230.34	Rha-C_12_-C_12_, Rha-C_12_-C_14:1_, Rha-C_12_-C_14_, Rha_2_-C_12_, Rha_2_-C_12_-C_12:2_
C_14:2_	7.59	0.09	C_15_H_26_O_3_	254.36	Rha-C_14:2_
C_14:1_	7.63	0.15	C_15_H_28_O_3_	256.38	Rha-C_12_-C_14:1_
C_14:0_	7.68	8.18	C_15_H_30_O_3_	258.40	Rha-C_12_-C_14_

The HPLC-MS spectra of the RL-SS14 in positive electrospray ionization (+ESI) mode (Fig. S4 of Supplementary data) revealed a total of 17 constituent rhamnolipid congeners (Table 3). The congeners were identified based on the results of the GC–MS analysis, calculations of elemental composition, and further comparison with available literature [21,32,33]. The predominant congener was identified as Rha-C_8_-C_10_ based on the relative abundance.

**Table 3:** Congeners of rhamnolipid (RL-SS14) produced by *Pseudomonas aeruginosa* SS14 using glycerol as the carbon source. The congeners were identified by HPLC-ESI-MS conducted in the positive mode. (Rha – Rhamnose moiety, FA – fatty acid).

RL type	RL homologue	Molecular		Detected ion	m/z	Relative abundance (%)
		Formula	Weight (M)			
Mono (Rha-FA)	Rha-C_8:2_	C_14_H_22_O_7_	302.32	[M+H]^+^	303	6.85
	Rha-C_9:2_	C_15_H_24_O_7_	316.35	[M-H+2Na]^+^	361	5.07
	Rha-C_10:2_	C_16_H_26_O_7_	330.37	[M+H]^+^	331	14.37
	Rha-C_10:1_	C_16_H_28_O_7_	332.39	[M + K]^+^	371	11.36
	Rha-C_12:2_	C_18_H_30_O_7_	358.43	[M+H]^+^	359	35.81
	Rha-C_14:2_	C_20_H_34_O_7_	386.48	[M-H+2Na]^+^	431	6.38
Mono (Rha-FA-FA)	Rha-C_8_-C_10_	C_24_H_44_O_9_	476.60	[M + Na]^+^ [M-H+2Na]^+^	499 521	18.41 26.31
	Rha-C_10_-C_12:2_	C_28_H_48_O_9_	528.67	[M-H+2Na]^+^	573	6.38
	Rha-C_12_-C_12_	C_30_H_56_O_9_	560.76	[M + K]^+^	599	3.47
	Rha-C_12_-C_14:1_	C_32_H_58_O_9_	586.80	[M+H]^+^	587	5.54
	Rha-C_12_-C_14_	C_32_H_60_O_9_	588.81	[M + Na]^+^ [M + K]^+^	589 627	5.17 10.23
Di (Rha_2_-FA)	Rha_2_-C_10_ Rha_2_-C_12_	C_22_H_40_O_11_ C_24_H_44_O_11_	480.54 508.60	[M+H]^+^	481	7.42
				[M + K]^+^	547	3.20
Di (Rha_2_-FA-FA)	Rha_2_-C_8_-C_10:1_	C_30_H_52_O_13_	620.72	[M+H]^+^	621	3.67
	Rha_2_-C_8_-C_10_	C_30_H_54_O_13_	622.74	[M+H]^+^	623	12.01
	Rha_2_-C_10_-C_10_	C_32_H_58_O_13_	650.79	[M + Na]^+^	683	9.42
	Rha_2_-C_12_-C_12:2_	C_36_H_62_O_13_	702.87	[M + K]^+^	741	10.91

### 3.3 Inhibition of biofilm formation

**Fig. 1:**
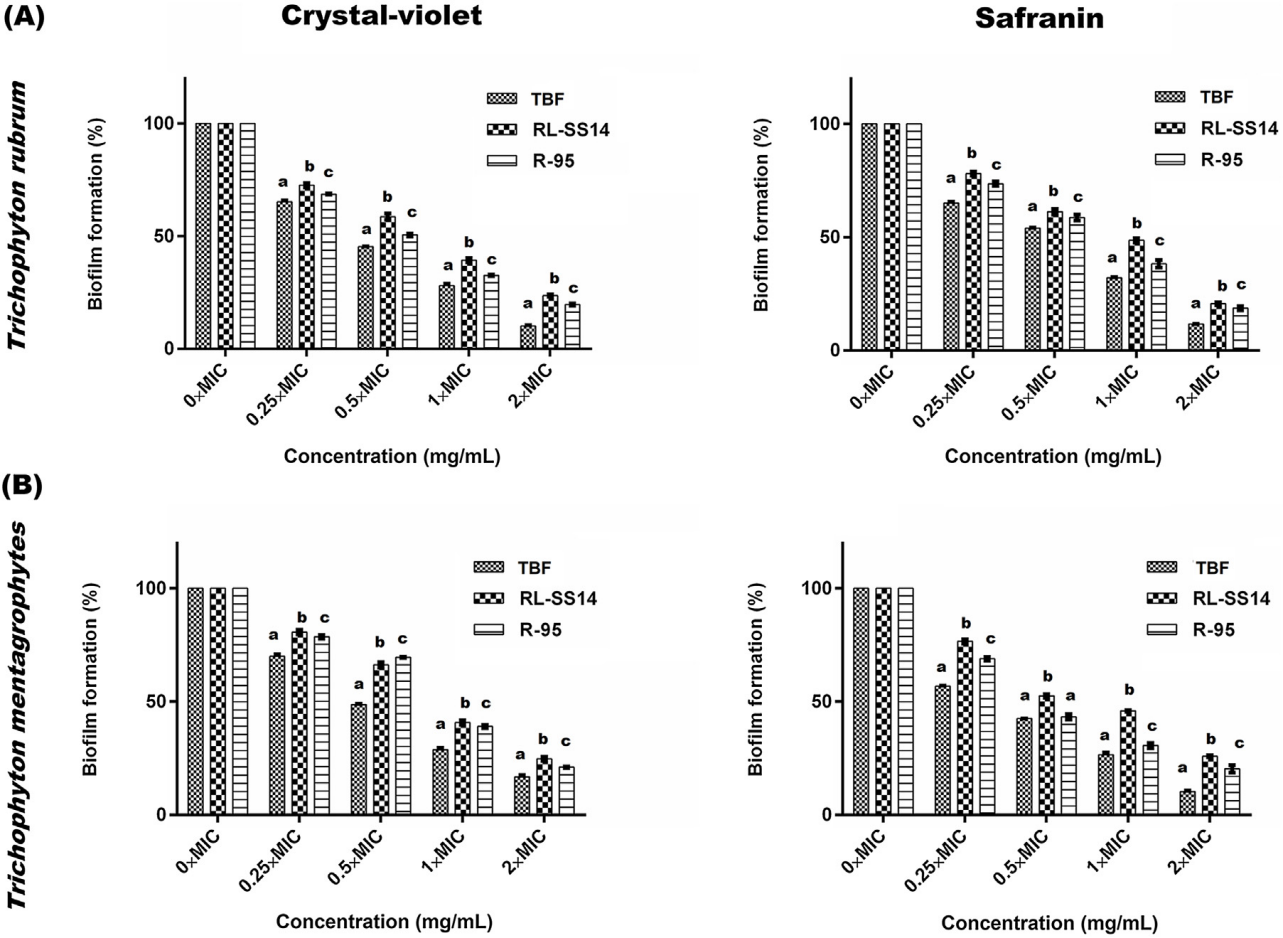
Inhibition of biofilm formation of *Trichophyton rubrum* and *Trichophyton mentagrophytes* on treatment with a rhamnolipid produced by *Pseudomonas aeruginosa* SS14 (RL-SS14) as determined by crystal violet (CV) and safranin staining. Commercial rhamnolipid (R-95) and terbinafine (TBF) were used as reference standards. Results are mean ± standard deviation (SD) and obtained from three independent experiments. Three replicate wells per condition were used. Different letters within each concentration indicate significantly different values as per the least significant difference (LSD) test.

The compounds were found to significantly inhibit the biofilm formation of *T. rubrum* and *T. mentagrophytes* (Fig. 1) as observed by CV (F_4,70_ = 671.044, *P* <  0.05 for *T. rubrum*; F_4,70_ = 495.036, *P* <  0.05 for *T. mentagrophytes*) and safranin (F_4,70_ = 717.186, *P* <  0.05 for *T. rubrum*; F_4,70_ = 408.868, *P* <  0.05 for *T. mentagrophytes*) staining. The compounds depicted a dose-dependent effect with the highest activity observed at 2 × MIC concentrations against both the pathogens (P < 0.05 in all treatment groups). Similar observations were also noted in the TBF treated samples. The results obtained with CV staining could be correlated with safranin staining for inhibition of biofilm formation (for RL-SS14, r = 0.98 and 0.97 against *T. rubrum* and *T. mentagrophytes* respectively).

### 3.4 Biofilm dispersal

**Fig. 2:**
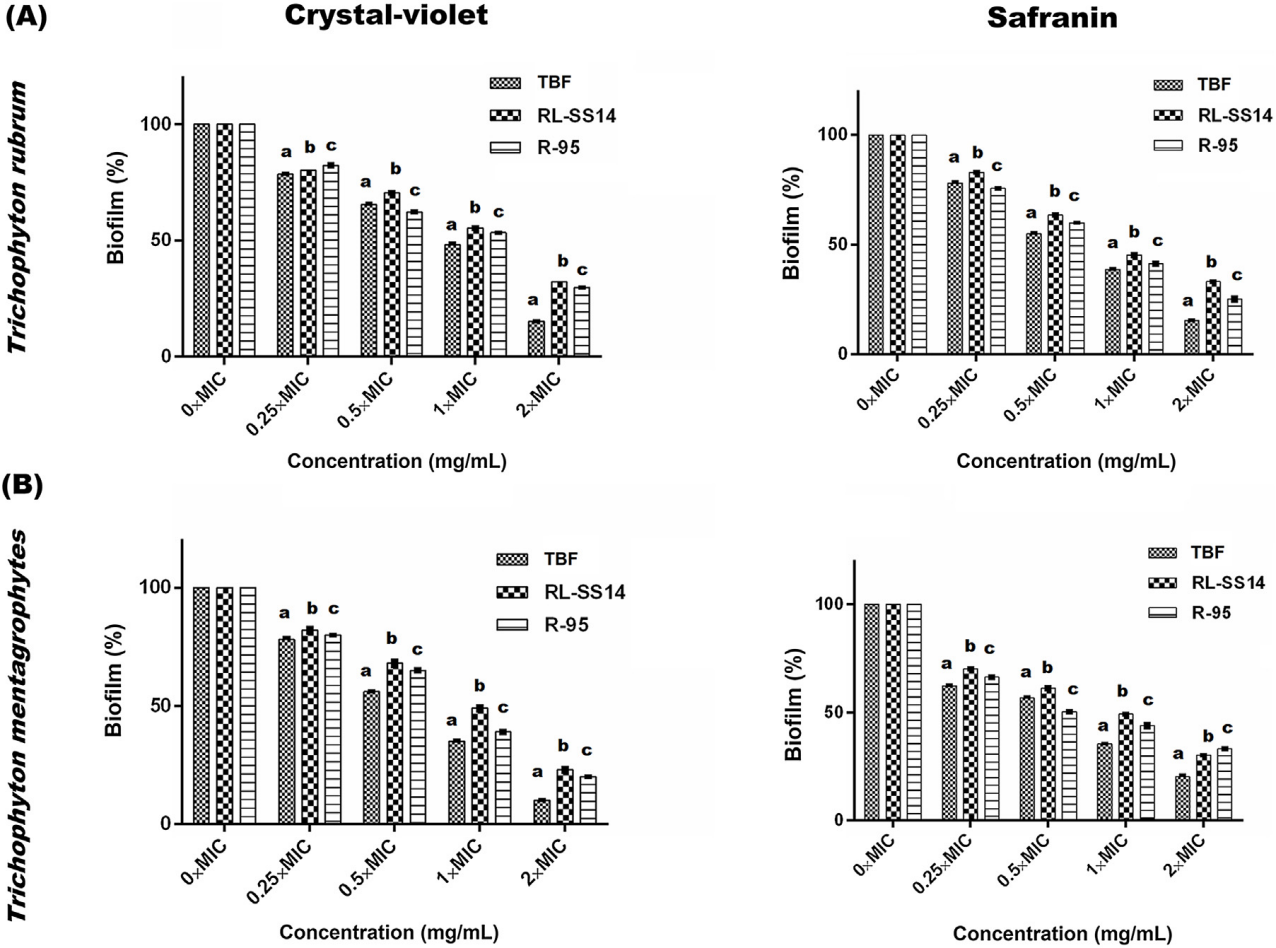
Effect of rhamnolipid produced by *Pseudomonas aeruginosa* SS14 (RL-SS14) on pre-formed biofilms of *Trichophyton rubrum* and *Trichophyton mentagrophytes* as determined by crystal violet (CV) and safranin staining. Commercial rhamnolipid (R-95) and antifungal terbinafine (TBF) were used as reference standards. Results are mean ± standard deviation (SD) and obtained from three independent experiments. Three replicate wells per condition were used. Different letters within each concentration indicate significantly different values as per the least significant difference (LSD) test.

On testing the effect of the antifungals against the mature biofilms, it was found that RL-SS14 had a disruptive impact on the biofilms of both *T. rubrum* (F_4,70_ = 613.332, *P* <  0.05) and *T. mentagrophytes* (F_4,70_ = 559.406, *P* <  0.05) in a concentration-dependant manner (Fig. 2) similar to TBF and R-95. The effect of the antifungals was measured as biofilm (%) at different concentrations ranging from 0.25 × MIC to 2 × MIC. The density of polysaccharides decreased significantly with the increase in the concentration of the antifungals (F_4,70_ = 795.830, *P* <  0.05 for *T. rubrum*; F_4,70_ = 383.242, *P* <  0.05 for *T. mentagrophytes*) as evident from the safranin staining of the polysaccharides and the EPS.

### 3.5 Microscopic evaluation of the biofilm disruption ability of RL-SS14

**Fig. 3:**
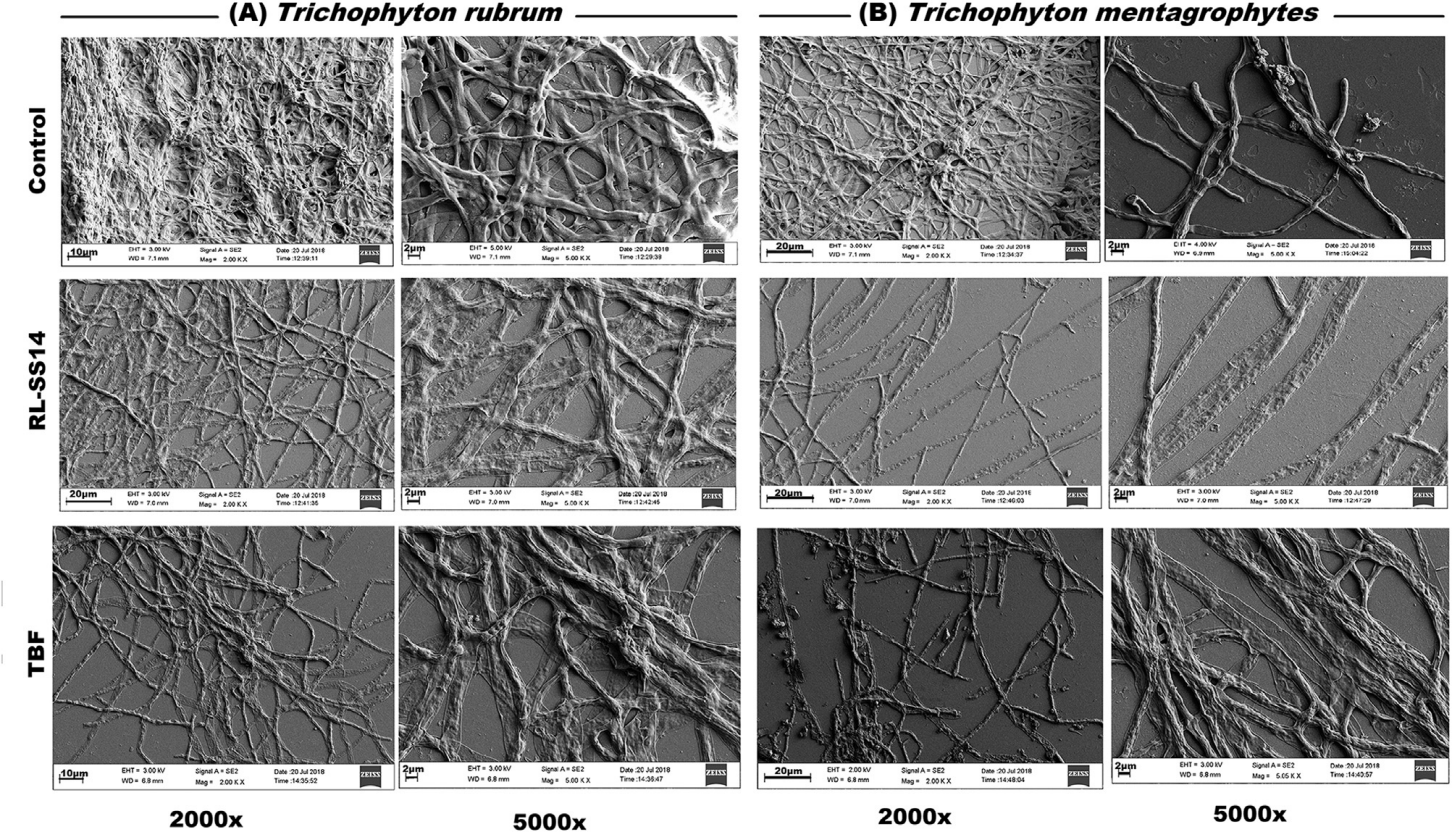
Representative scanning electron microscopic (SEM) images of the effect of rhamnolipid produced by *Pseudomonas aeruginosa* SS14 (RL-SS14) and Terbinafine (TBF) on mature biofilms of *Trichophyton rubrum* and *Trichophyton mentagrophytes*. The samples were treated at 2 × MIC of RL-SS14 (1 mg/mL for *T. rubrum* and 0.25 mg/mL for *T. mentagrophytes*) or TBF (0.03 mg/mL for *T. rubrum* and 0.062 mg/mL for *T. mentagrophytes*). The morphological changes were visualized and compared to the untreated control samples. Scale bar is 20 μm (2000×) and 2 μm (5000×).

**Fig. 4:**
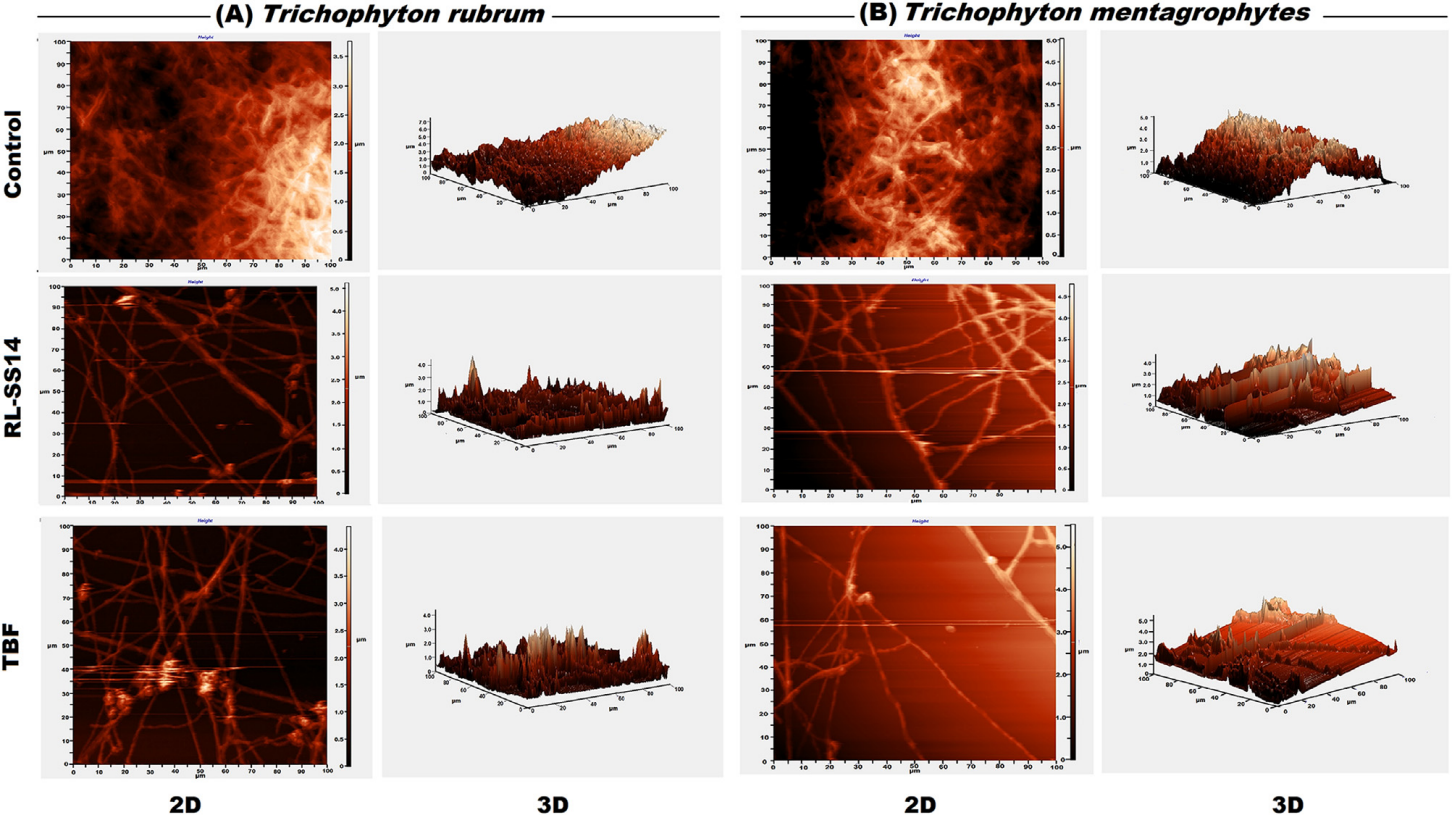
Representative two and three-dimensional atomic force microscopic (AFM) images of the effect of rhamnolipid produced by *Pseudomonas aeruginosa* SS14 (RL-SS14) on mature biofilms of *Trichophyton rubrum* and *Trichophyton mentagrophytes*. Samples treated with terbinafine (TBF) served as the standard drug control. The topographical changes in the biofilms induced on treatment with 2 × MIC of RL-SS14 (1 mg/mL for *T. rubrum* and 0.25 mg/mL for *T. mentagrophytes*) and TBF (0.03 mg/mL for *T. rubrum* and 0.062 mg/mL for *T. mentagrophytes*) were visualized and compared to the untreated control samples.

**Fig. 5:**
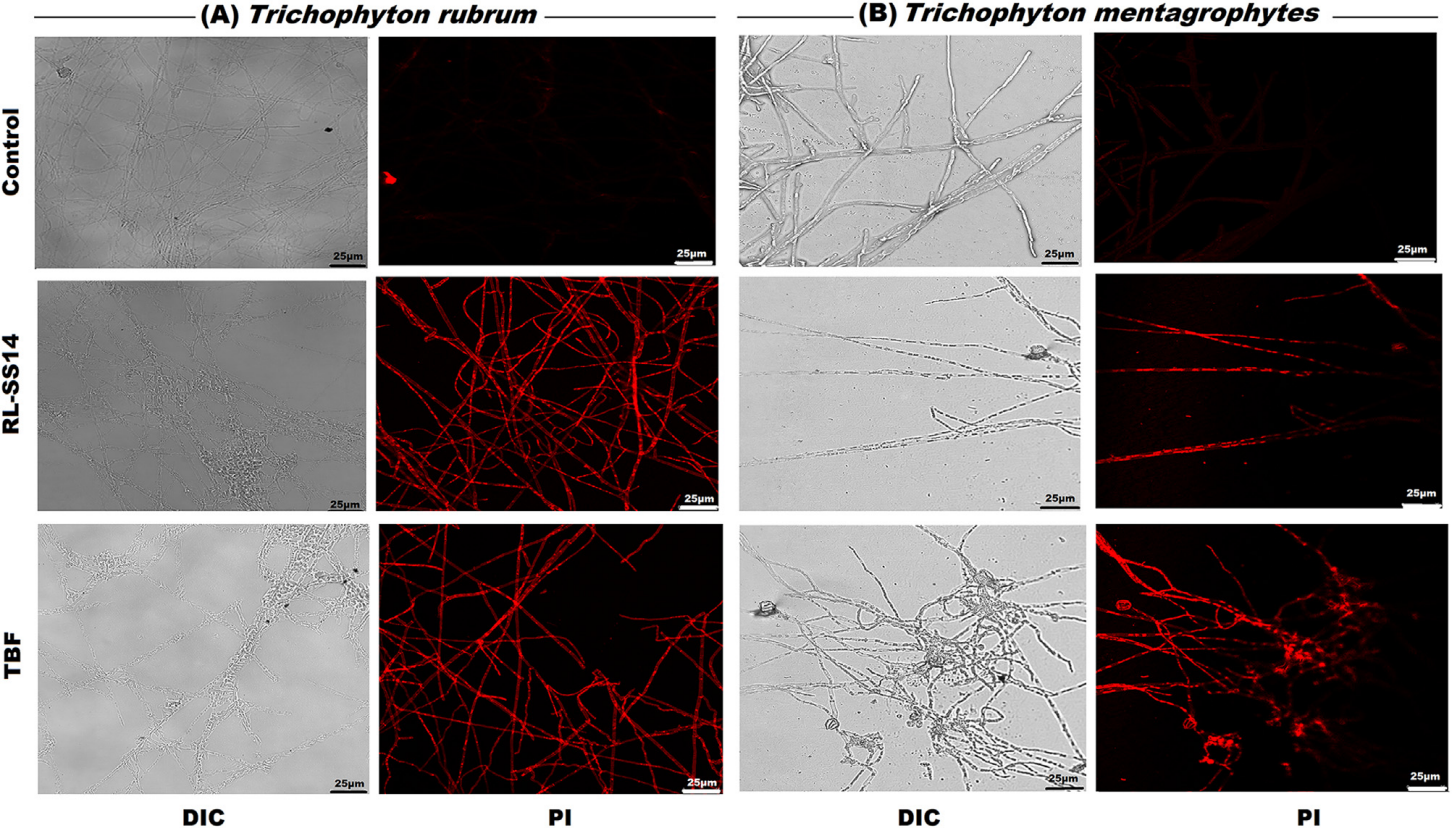
Representative images obtained after confocal laser scanning microscopy (CLSM) of the biofilms of *Trichophyton rubrum* and *Trichophyton mentagrophytes* stained with propidium iodide (PI) after exposure to 2 × MIC of a rhamnolipid produced by *Pseudomonas aeruginosa* SS14 (RL-SS14; 1 mg/mL for *T. rubrum* and 0.25 mg/mL for *T. mentagrophytes*) or Terbinafine (TBF; 0.03 mg/mL for *T. rubrum* and 0.062 mg/mL for *T. mentagrophytes*). Untreated samples served as a negative control. Scale bar is 25 μm.

Electron, atomic force, and confocal microscopic evaluations were performed to visualize the biofilm dispersal capacity of RL-SS14 against mature biofilms of *T. rubrum* and *T. mentagrophyte*s, (Fig. 3, Fig. 4, Fig. 5). Observations revealed that the fungal biofilms treated with RL-SS14 and TBF at 2 × MIC varied distinctly in structure and spatial arrangement in comparison to the untreated control of both *T. rubrum* and *T. mentagrophytes*. SEM micrographs of the colony surface of the biofilms (Fig. 3**a** and **b**) revealed distinct biofilm architecture with ECM in the control samples. On treatment for 24 h, the morphological arrangement of the hyphae was noticeably different with substantially reduced ECM, damaged hyphae with flattened structures, and less densely packed mycelia.

Three-dimensional AFM images (Fig. 4**a** and **b**) revealed that *T. rubrum* and *T. mentagrophytes* biofilms after treatment with RL-SS14 and TBF exhibited severe changes in biofilm topography and architecture as compared to the control. Treated biofilms showed lesser mycelia with reduced height (Z axes) in comparison to the untreated samples, which had a uniform arrangement of the filamentous cells. The AFM findings corroborated the SEM observations of damage to the cell morphology resulting in height differences seen post-treatment.

Viability assessment of the treated samples in comparison to the untreated control by PI staining as visualized in a confocal microscope (Fig. 5**a** and **b**) revealed increased fluorescence due to uptake of the dye and hence, signifying cell death. The untreated control samples of *T. rubrum* and *T. mentagrophytes* exhibited no fluorescence depicting healthy cells.

## 4 Discussion

Biofilms are highly resistant to several antimicrobial agents and offer significant advantages to fungi over any other mode of propagation. Consequently, biofilms constitute an escalating problem in the context of human health [34]. The efficiency of RLs against bacterial and yeast biofilms has been evaluated in several studies [23,29,35]. However, anti-biofilm activities of RLs against filamentous fungal species are rarely reported. Based on our previous findings demonstrating the inhibitory effect of RL-SS14 against the planktonic cells of *T. rubrum* [25] and the fact that dermatophytic infections are frequently related to biofilm formation, the current study investigates the effect of RL-SS14 against the sessile forms of *T. rubrum* and *T. mentagrophytes*.

Screening of the *in vitro* antifungal activity can be useful to detect resistance trends and the capacity of a prospective antimicrobial to eradicate a specific fungal species [36]. The antifungal activities of RL-SS14 samples produced using different carbon sources were different, which can be explained by the variation in the RL congeners influenced by the type of substrates used for production, as reported previously by others [37,38]. In our study, RL-SS14 produced using glucose and glycerol exhibited the highest antifungal potentials against *T. rubrum* and *T. mentagrophytes*, respectively. In terms of composition, RL-SS14 produced using glucose contained mono-RLs only, whereas that produced using glycerol was a mixture of mono and di-RLs with a higher concentration of mono-RL congeners. Das et al. [37] reported an increase in the antimicrobial action with an increase in the proportion of mono-RL congeners. They hypothesized that as the concentration of mono-RL increases over di-RL, the hydrophobicity of the RL mixture increasesfavouring its permeation through membrane lipids. Also, the activity of RLs varies from pathogen to pathogen, depending on their interaction with the target organism based on cell wall composition. This might also be a determining factor in the overall antifungal effect of an RL sample in addition to the proportion of mono and di-RL congeners.

BS, including RLs, affect the initial attachment on various surfaces by altering the cell surface hydrophobicity, thereby preventing biofilm formation in bacteria and fungi [14]. On treatment with RL-SS14, significant concentration-dependant inhibition of biofilm biomass (CV staining) and extracellular matrix (ECM; safranin staining) of *T. rubrum* and *T. mentagrophytes* was observed to varying extents between 0.25–2 × MICs. ECM production occurs during biofilm growth and is responsible for the mechanical stability of biofilms and is instrumental in delaying the penetration of drugs rendering resistance [29]. Hence, in this study, the ability of RL-SS14 to affect the ECM development of the test pathogens was determined. A plausible explanation for the inhibitory effect of RL-SS14 against biofilm formation seems to be its antifungal property and alteration of the cell surface hydrophobicity influencing the adhesion of the fungi to form biofilms, as has been reported for other RLs. Al-Tahhan et al. [39] observed that RLs cause the loss of overall cellular fatty acid content, lipopolysaccharides in particular, thereby increasing the cell surface hydrophobicity. Thus, the biofilm inhibition activity of RLs depends on the alteration of the physical properties of the membrane of the pathogen. Hence, the probability of the development of resistance through spontaneous mutations is lower [35].

Mature biofilms are especially problematic to get rid of and hence, are more relevant from a medical perspective [2]. RL-SS14 could progressively eradicate mature *T. rubrum,* and *T. mentagrophytes* generated biofilms at concentrations ranging from sub-MIC to 2-fold of the MIC. It is well-documented that the antifungal drug concentrations required to reduce metabolic activity by 50 % are higher for biofilms than for planktonic cells [40]. Dusane and his co-workers [41] reported a similar trend in a RL produced by a marine strain of *Serratia marcescens*, which exhibited significant anti-adhesive and biofilm disruptive ability against *C. albicans* at a concentration which was 4-fold of the MIC.

The ability of RL-SS14 to disrupt mature biofilms of the pathogens was further visualized by employing imaging techniques (SEM, AFM, and CLSM). SEM and AFM images of RL-SS14 treated biofilms on glass coverslips showed marked disruption of the biofilm matrix, confirming the results obtained in the safranin and CV assays. Mycelial distortion with flattened hyphae and reduced mycelial height due to exudation of cellular contents were noticed, indicating that RL-SS14 disrupts the fungal cell wall. The observed mycelial effects might be a result of the substantial reduction in ECM and loosely arranged mycelia on exposure to RL-SS14, rendering the hyphae susceptible to the membrane permeabilizing effects of the BS, as reported earlier [13]. Furthermore, CLSM micrographs with PI staining substantiates the spectroscopic and ultramicroscopic observations that RL-SS14 could penetrate the ECM of the fungi making the cells sensitive to its membrane permeabilization action, decreasing its viability and causing cell death.

In conclusion, RL-SS14 demonstrated promising anti-biofilm properties. To the best of our knowledge, this is the first report describing the anti-biofilm effect of rhamnolipids against dermatophytic biofilms. Our results described the inhibitory and disruptive effects of RL-SS14 against *T. rubrum,* and *T. mentagrophytes* generated biofilms. A detailed analysis of the mechanism of action of the test biosurfactant and *in vivo* studies are needed in the future for the establishment of RL-SS14 as an alternative antifungal to treat recalcitrant dermatophytic infections.

## Funding

This study was supported by the institutional core budget of the Institute of Advanced Study in Science and Technology (IASST), Dept. of Science and Technology (DST), Govt. of India.

## CRediT authorship contribution statement

**Suparna Sen:** Conceptualization, Methodology, Validation, Formal analysis, Investigation, Data curation, Writing - original draft, Writing - review & editing, Visualization. **Siddhartha Narayan Borah:** Methodology, Validation, Formal analysis, Data curation, Writing - original draft, Writing - review & editing. **Arijit Bora:** Writing - review & editing, Supervision. **Suresh Deka:** Resources, Writing - review & editing, Funding acquisition, Supervision.

## Declaration of Competing Interest

The authors report no declarations of interest.
