# Supplementary material for: Rhamnolipid exhibits anti-biofilm activity against the dermatophytic fungi Trichophyton rubrum and Trichophyton mentagrophytes
Source: Biotechnol Rep (Amst). 2020 Aug 19;27:e00516. doi: 10.1016/j.btre.2020.e00516 (PMC7451867; doi:10.1016/j.btre.2020.e00516)
Supplement: Supplementary file 1 [file mmc1.pdf]

Supplementary Data

Rhamnolipid exhibits anti-biofilm activity against the dermatophytic fungi *Trichophyton rubrum* and *Trichophyton mentagrophytes*

Suparna Sen[a], Siddhartha Narayan Borah[a,b], Arijit Bora[c], Suresh Deka[a]

[a]Environmental Biotechnology Laboratory, Life Sciences Division, Institute of Advanced Study in Science and Technology, Guwahati, India

[b]Centre for the Environment, Indian Institute of Technology Guwahati, Guwahati, India

[c]Department of Bioengineering and Technology, Institute of Science and Technology, Gauhati University, Guwahati, India

*Corresponding author.

E-mail: sureshdeka@gmail.com (Suresh Deka)

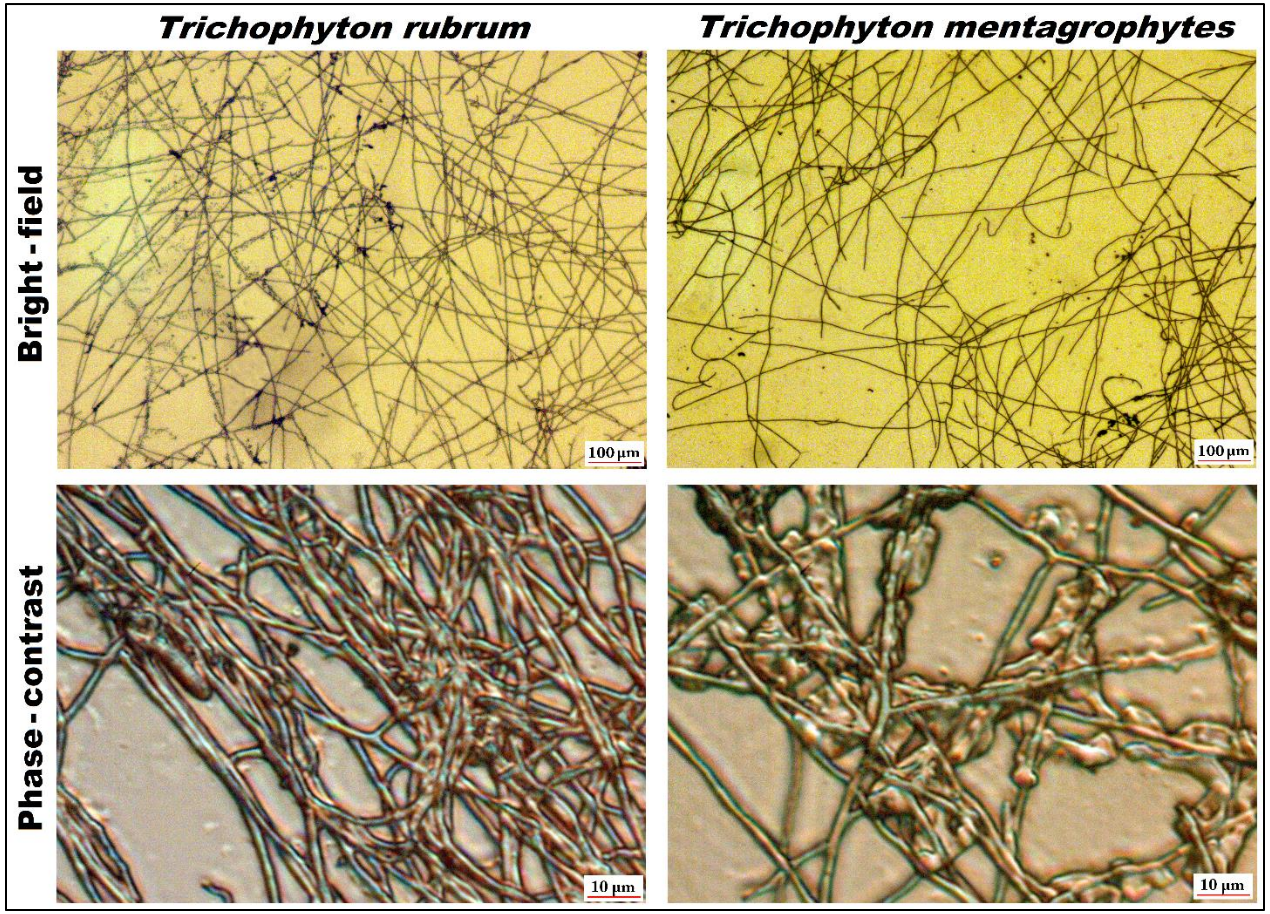

Fig. S1. Light micrographs of the biofilms formed by *Trichophyton rubrum* and *Trichophyton mentagrophytes* on glass coverslips in RPMI-1640 medium after 96 h of incubation at 28° C. Bright-field images (10× magnification) exhibit the mycelial proliferation over the glass coveslip, whereas the phase-contrast images (100× magnification) reveal the extracellular matrix around the fungal mycelia.

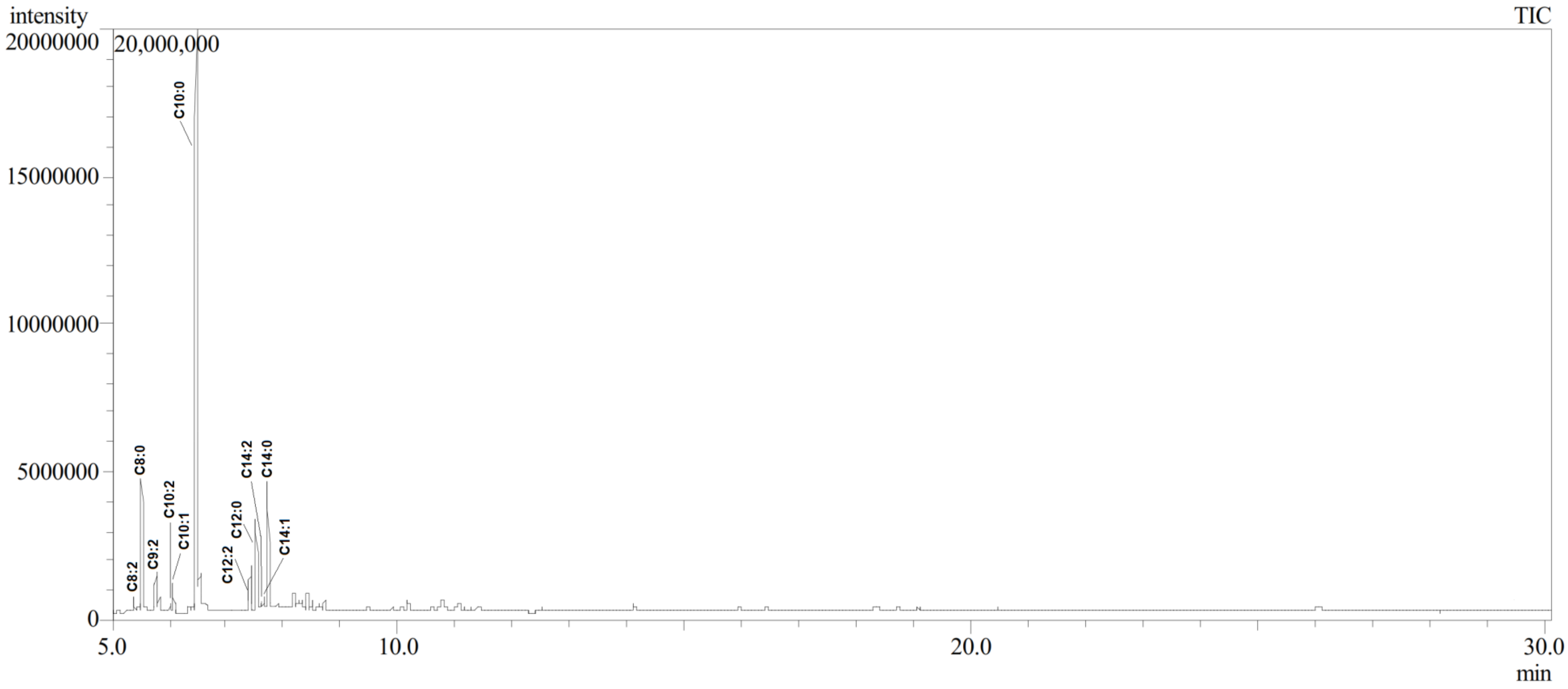

Fig. S2. Gas chromatogram of the rhamnolipid produced by *Pseudomonas aeruginosa* SS14 using glycerol as the sole source of carbon. The rhamnolipid sample was derivatized to obtain 3-OH fatty acid methyl esters (3-OH FAMEs) of the lipidic components of the constituent congeners.

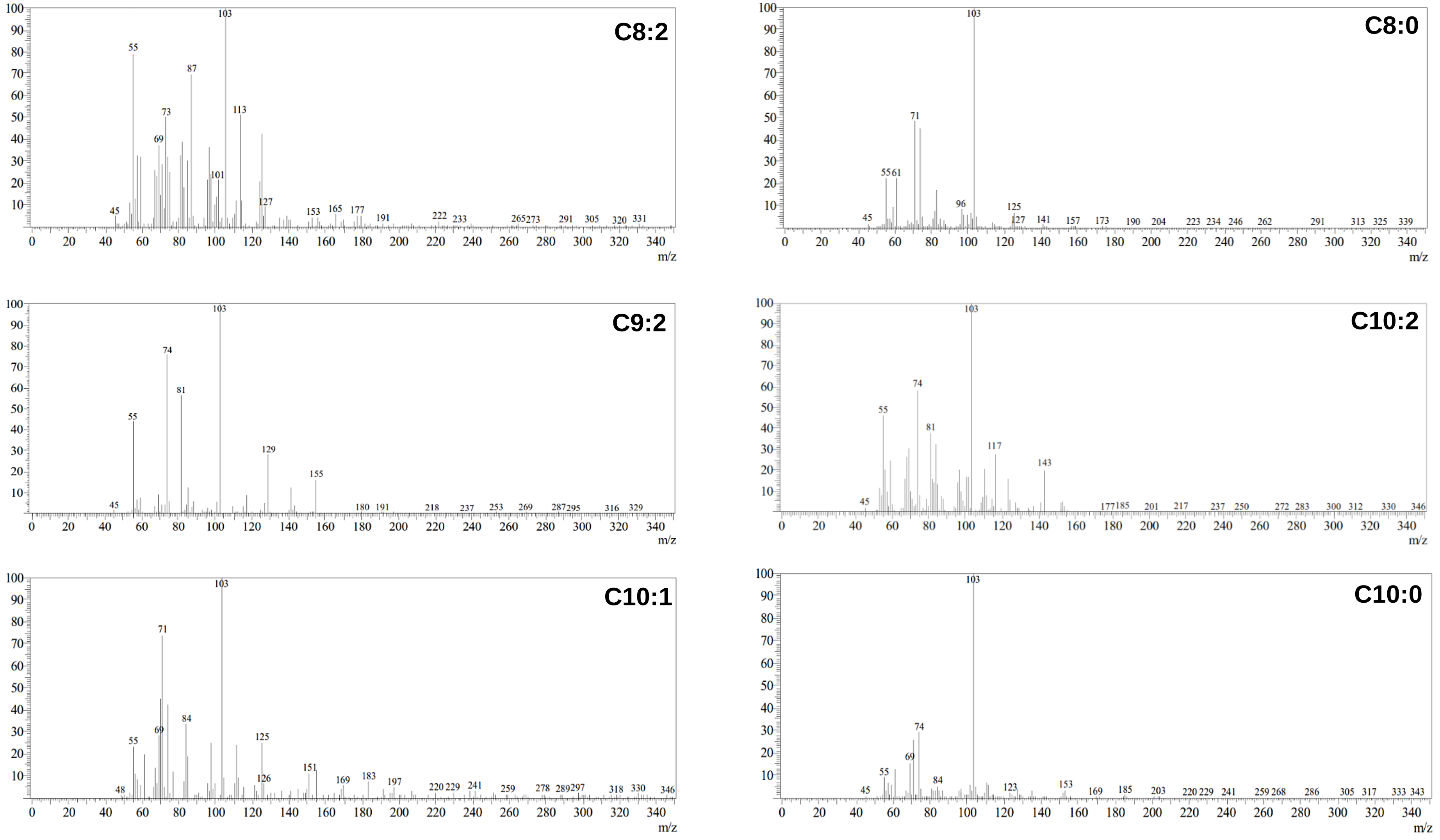

Fig. S3. Mass spectra of each individual FAMEs obtained in the GC analyses. The peak corresponding to m/z 103 is the characteristic fragment of 3-OH FAMEs (continued on next page).

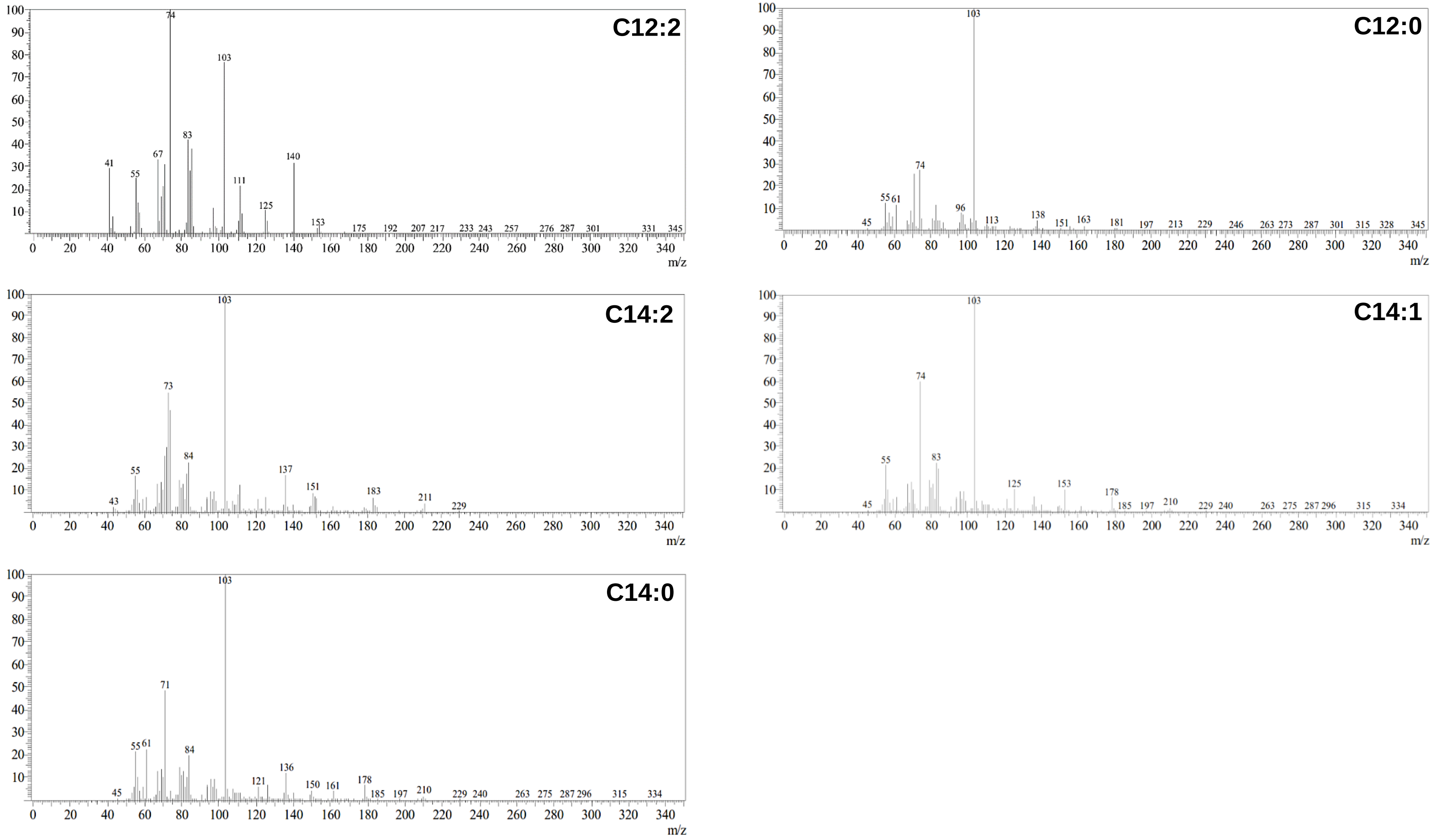

Fig. S3. (continued from the previous page) Mass spectra of each individual FAMEs obtained in the GC analyses. The peak corresponding to m/z 103 is the characteristic fragment of 3-OH FAMEs.

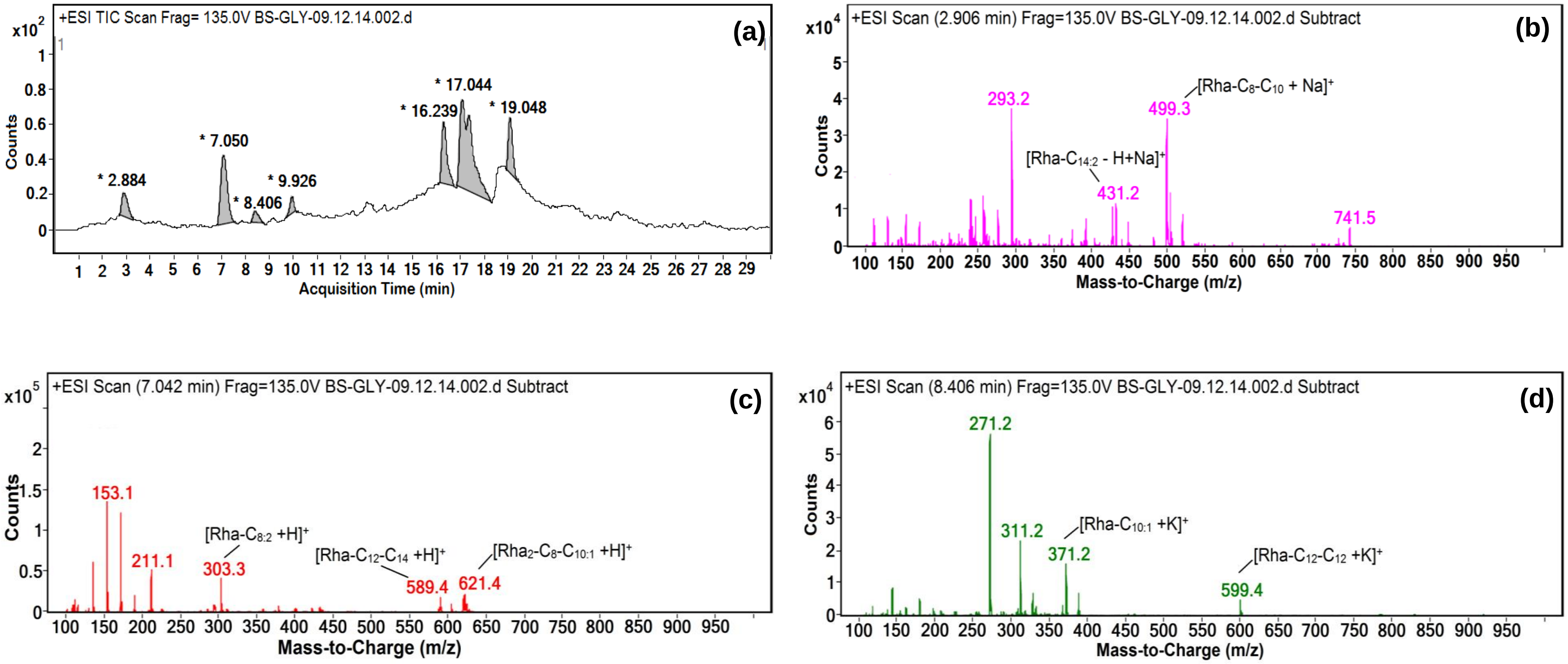

Fig. S4. HPLC-ESI-MS analysis of the rhamnolipid produced by *Pseudomonas aeruginosa* SS14 using glycerol as the sole source of carbon. **(a)** Total ion chromatogram. **(b), (c), and (d)** Mass spectra of the fractions (continued on the next page).

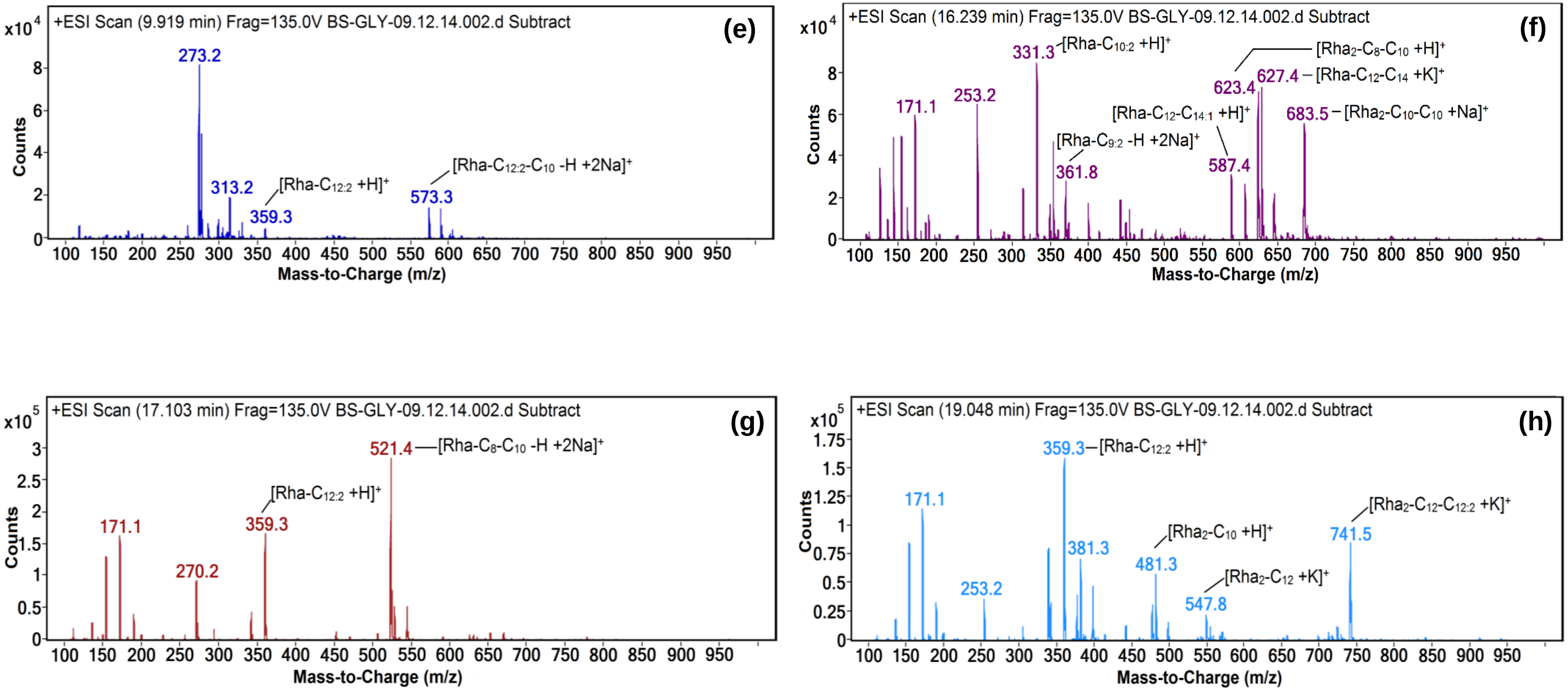

Fig. S4. (continued from the previous page) HPLC-ESI-MS analysis of the rhamnolipid produced by *Pseudomonas aeruginosa* SS14 using glycerol as the sole source of carbon. **(e), (f), (g), and (h)** Mass spectra of the fractions.
